# Timing and probability of arrival for sea lice dispersing between salmon farms

**DOI:** 10.1098/rsos.220853

**Published:** 2023-02-08

**Authors:** Peter D. Harrington, Danielle L. Cantrell, Michael G. G. Foreman, Ming Guo, Mark A. Lewis

**Affiliations:** ^1^ Department of Mathematical and Statistical Sciences, University of Alberta, Edmonton, Alberta, Canada; ^2^ Department of Biological Sciences, University of Alberta, Edmonton, Alberta, Canada; ^3^ California Department of Fish and Wildlife, Marine Region’s Fisheries Analytics Project, 20 Lower Ragsdale Drive, Suite 100, Monterey, CA 93940, USA; ^4^ Institute of Ocean Sciences, Fisheries and Oceans Canada, Sidney, British Columbia, Canada

**Keywords:** arrival time, sea lice, salmon farms, dispersal, bio-physical model

## Abstract

Sea lice are a threat to the health of both wild and farmed salmon and an economic burden for salmon farms. With a free-living larval stage, sea lice can disperse tens of kilometres in the ocean between salmon farms, leading to connected sea louse populations that are difficult to control in isolation. In this paper, we develop a simple analytical model for the dispersal of sea lice (*Lepeophtheirus salmonis*) between two salmon farms. From the model, we calculate the arrival time distribution of sea lice dispersing between farms, as well as the level of cross-infection of sea lice. We also use numerical flows from a hydrodynamic model, coupled with a particle tracking model, to directly calculate the arrival time of sea lice dispersing between two farms in the Broughton Archipelago, British Columbia, in order to fit our analytical model and find realistic parameter estimates. Using the parametrized analytical model, we show that there is often an intermediate interfarm spacing that maximizes the level of cross-infection between farms, and that increased temperatures will lead to increased levels of cross-infection.

## 1. Introduction

Marine populations are often connected over large distances due to larval dispersal. Once thought to be open populations with continuous exchanges of larvae, it is now understood that many marine populations depend directly on the degree of larval exchange between population patches, and that these connected patches act as metapopulations [1–3]. The degree of connectivity between habitat patches in a metapopulation is a function of many variables, including the strength of the ocean currents on which the larvae depend to disperse and the environmental conditions of the ocean which impact biological processes such as maturation and survival. Research into larval dispersal in marine metapopulations has led to a greater understanding of the population dynamics of corals [4], coral reef fish [5] and sea turtles [6], as well as the efficacy of Marine Protected Areas [7,8]. It has also been used to determine the level of sea lice dispersal between salmon farms and the effect of coordinated treatment plans in salmon farming regions [9–12].

**Figure 1.**
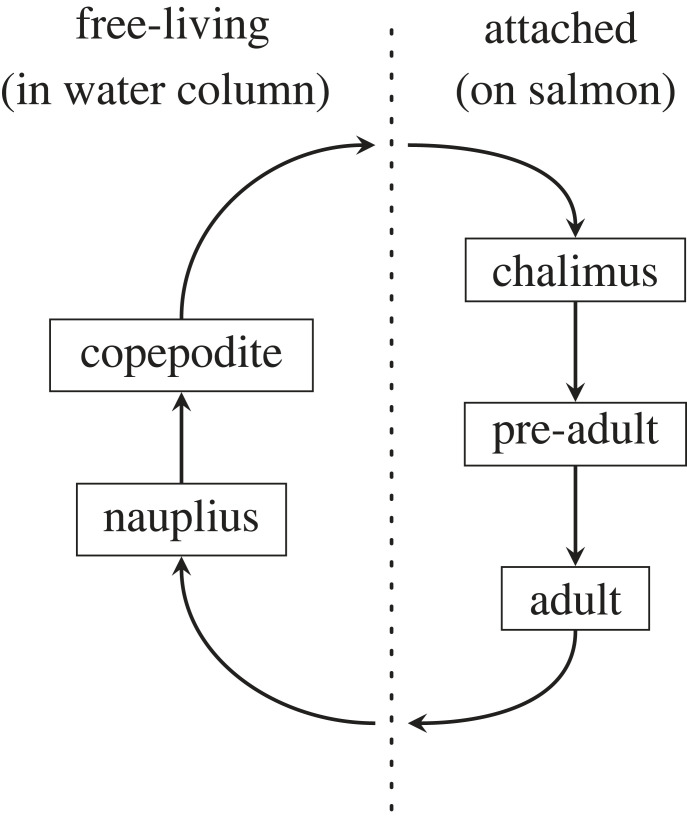
A simplified schematic of the life cycle of the sea louse, *Lepeophtheirus salmonis*. The attached stages live on wild or farmed salmon and the free-living stages disperse in the water column. Larvae must mature through the nauplius stage into the copepodite stage before they are able to attach to a salmonid host. In this paper, we focus on the time that it takes for sea lice in the free-living stages to disperse in the water column from one salmon farm to another.

Sea lice (*Lepeophtheirus salmonis*) are parasitic marine copepods that feed on the epidermal tissues, muscle and blood of salmon [13]. A free-living nauplius stage allows sea lice to disperse tens of kilometres in the ocean while developing into infectious copepodites that can attach to their salmonid hosts, on which sea lice complete the remainder of their life cycle (see figure 1) [14,15]. High infestation levels on farmed salmon have been shown to lead to mortality and morbidity [16], and lesions and stress from infestations make salmon susceptible to secondary infections, which have led to large economic consequences for the salmon farming industry [17]. On wild juvenile salmon, infestation with sea lice can lead to mortality [18] or other physiological [19] and behavioural effects [20,21]. In near coastal areas, elevated levels of sea lice from salmon farms have been detected on juvenile salmon up to tens of kilometres away and these high levels of infection have contributed to population level declines in pink salmon [18,22–24].

In dense salmon farming regions such as Norway, Scotland and Canada there is evidence that sea lice populations on salmon farms are connected via larval dispersal, therefore acting as connected metapopulations, though the degree of larval dispersal between farms may not always be solely based on distance [10,11,25–27]. Evidence for connectivity has been found by tracking sea lice particles in hydrodynamic models [9–11,26] and by fitting metapopulation models to data of sea lice counts on salmon farms [25,27]. In Norway, where most salmon farms are located in fjords along the coast, seaway distance has been used as a simple measure of farm connectivity over a large scale [25]. However, in more complex coastal areas there is evidence that the highest density of infectious sea lice may not always be centred around the farm of origin, as local currents can move larvae away from the farm before they become infectious. Using a hydrodynamic model, Cantrell *et al.* [10] found that the density of infectious copepodites from the five south-easterly farms in the Broughton Archipelago, British Columbia, was highest further than 15 km away from the nearest farm. Less drastically, using a mechanistic model fit to empirical data, Peacock *et al.* [24] found that the highest density of copepodites was a few kilometres away from each farm along a wild salmon migration corridor, also in the Broughton Archipelago.

The degree of interfarm connectivity can have large consequences for treating for sea louse outbreaks. Theoretical analyses have shown that when salmon farms are connected and treatment is applied once sea lice counts reach a certain threshold, coordinated treatment between farms leads to less frequent treatment overall whereas uncoordinated treatment between farms requires more frequent treatment overall and can lead to unpredictable sea louse population dynamics [28]. Calculating the probability of sea lice dispersing to other farms is integral in determining which farms may be the largest sources of sea lice spread in a salmon farming region and thus which may be driving spread [10,29]. Lastly, and perhaps most importantly, determining the probability of sea lice arrival onto other farms is critical in understanding where to place farms in a salmon farming region, or which to first remove.

To date, most of the research into the degree of connectivity between salmon farms has been either region specific, using hydrodynamical models [9,10], or at a large scale, with statistical analyses [25,30]. Hydrodynamic models can be very useful in determining connectivity in the specific regions for which they are run as they provide detailed trajectories of sea lice particles leaving each salmon farm. However, results from these specific regions may be difficult to generalize to others if current patterns or environmental variables affecting sea louse survival and maturation differ. Conversely, statistical analyses are useful for determining the broad drivers of spread as they can uncover the general underlying relationships between different variables and farm connectivity. However, they often lack sufficient detail on local current patterns to allow for detailed investigations into how certain parameter interactions affect the degree of connectivity between two farms. Thus, there are still many general questions surrounding interfarm connectivity that requires new approaches to investigate.

In this paper, we are interested in understanding how the distance (or ‘spacing’) between two farms affects the probability that a sea louse (*L. salmonis*) travels from one of those farms to the other (‘cross-infection’). Specifically, we aim to answer the following questions:

(i) How does the degree of cross-infection depend on the spacing between farms?
(ii) Are there scenarios where cross-infection between farms is maximized at an intermediate farm spacing, where farms are several kilometres apart?
(iii) Does the relationship between cross-infection and farm spacing change in systems dominated by advection (primarily driven by mean currents) versus diffusion (no mean currents)?
(iv) How does the maturation time for nauplii to develop into infectious copepodites affect cross-infection?

In order to answer these questions, we construct a mechanistic model that is both sufficiently simple to investigate analytically and numerically in detail, but sufficiently realistic to capture the essential components of ocean circulation and sea louse biology. For credible analysis the simple model is fitted to realistic sea lice particle tracking dispersal data to ensure accurate estimates of oceanic advection and diffusion as well as sea louse maturation times. The approach here is to use numerical flows from a three-dimensional computational hydrodynamic model, the finite volume community ocean model (FVCOM), along with a particle tracking model, to fit a simple one-dimensional analytical model that describes the movement of sea lice between two salmon farms in a channel in the Broughton Archipelago, British Columbia.

The paper is structured as follows. First, we use a simple mechanistic model of sea lice (*L. salmonis*) dispersing between two salmon farms to calculate the time it takes for sea lice leaving one farm to arrive on the other. We begin by presenting the analytical results for simple particles dispersing, ignoring the maturation required for sea lice to become infectious, before presenting the full arrival time density for sea lice that encompasses the non-infectious nauplius stage and infectious copepodite stage. Next, we calculate the arrival time directly using the numerical flows from FVCOM coupled with a particle tracking model to fit our simple mechanistic model and find parameter estimates. Lastly, we use our parametrized mechanistic model to investigate the questions (i)–(iv) surrounding cross-infection and farm placement and we show that cross-infection can be maximized when farms are spaced several kilometres apart due to the development time required for sea lice to become infectious.

## 2. Methods

### 2.1. Study area

**Figure 2.**
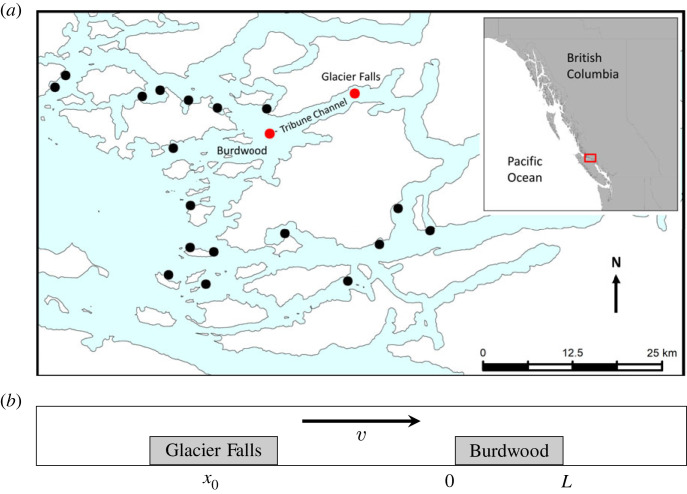
The two salmon farms that are used to calculate the time and probability of arrival for sea lice dispersing between farms. (*a*) A map of the Broughton Archipelago (red inset) in British Columbia. All active farms from 2009 are shown in black and the two farms used in this study highlighted in red. The release farm is the eastern farm, Glacier Falls, which is located in Tribune Channel and the receiving farm is the western farm, Burdwood. (*b*) The one-dimensional representation of Tribune Channel used in the mathematical analysis, where sea lice are released as a point source from Glacier Falls at position *x*_0_ and can arrive onto Burdwood between 0 and *L*, where *L* is the length of the Burdwood farm. Note that the position of the farms has been switched so the advective coefficient, *v*, is positive.

The Broughton Archipelago is a group of islands located between the northeast coast of Vancouver Island and the mainland of British Columbia (figure 2). The Broughton Archipelago has several active salmon farms and has been at the centre of the debate of the effect of sea lice on wild salmon [18,22,31–33]. The rivers in the Broughton are major migration routes for both pink (*Oncorhynchus gorbuscha*) and chum (*Oncorhynchus keta*) salmon, and sea lice from salmon farms in this region have contributed to population level declines in pink salmon [18]. Currently, certain salmon farms are being removed under a new agreement between the governments of British Columbia and the Kwikwasut’inuxw Haxwa’mis, ’Namgis and Mamalilikulla First Nations [34]. The abundance of sea louse data from counts on farmed and wild salmon as well as the complex hydrodynamical particle tracking simulations run for this region make the Broughton Archipelago an ideal area to investigate the cross-infection of sea lice between salmon farms.

### 2.2. Mechanistic model

In order to calculate the arrival time of sea lice travelling between salmon farms and the probability of arrival, we first need a model for how sea lice disperse along a channel and arrive at a salmon farm. For now, we shall ignore the maturation time required for newly released nauplii to develop into infectious copepodites in order to gain a comprehensive understanding of the arrival time distribution and to simplify the details of the initial mathematical analysis. Therefore, we are assuming that all sea lice released from a salmon farm are infectious and model their dispersal using the following advection–diffusion equation:

2.1 $$\frac{\partial }{\partial t}p(x,t)=-\underset{\mathrm{advection}}{\underbrace{\frac{\partial }{\partial x}(vp(x,t))}}+\underset{\mathrm{diffusion}}{\underbrace{\frac{\partial^{2}}{\partial x^{2}}(Dp(x,t))}}-\underset{\mathrm{mortality}}{\underbrace{\mu p(x,t)}}-\underset{\mathrm{arrival}\;\mathrm{onto}\;\mathrm{farm}}{\underbrace{h(x)\alpha p(x,t)}}$$

2.2 $$p(x,0)=\delta (x-x_{0})$$

2.3 $$\begin{array}{rl} \mathrm{and}\;h(x) & =\left\{ \begin{array}{cc} 1 & x\in [0,L]\\ 0 & \text{otherwise}, \end{array}\right.\; \end{array}$$

where *p*(*x*, *t*) is the density of sea lice at position x∈R and time *t* ≥ 0, *x*_0_ is the position of the initial farm, and the farm at which sea lice are arriving is located between [0, *L*], where *L* is the length of the farm. We use a delta function positioned at the initial farm, *x*_0_, as the initial condition because sea lice nauplii hatch directly from the egg strings of a gravid female into the water column [16]. The rate at which sea lice then arrive onto the receiving farm, through successful attachment to salmonid hosts, is given by *α*; the mortality rate of lice is *μ*; advection, which represents the general seaward flow due to river output, is given by *v*; and diffusion, representing mixing due to winds and tidal flow, is given by *D*. This equation has previously been used to model sea lice movement in the Broughton Archipelago, to demonstrate the increase in sea lice on wild salmon caused by salmon farms along salmon migration routes [22–24]. The necessary boundary conditions that accompany equation (2.1) are

2.4 $$\;\lim_{x\to -\infty }p(x,t)=0$$

2.5 $$\;\lim_{x\to \infty }p(x,t)=0$$

2.6 $$\lim_{x\to -\infty }\frac{\partial }{\partial x}p(x,t)=0$$

2.7 $$\;\mathrm{and}\;\;\lim_{x\to \infty }\frac{\partial }{\partial x}p(x,t)=0.$$

Looking at equation (2.1) we can see that there are two ways that sea lice can stop dispersing and are removed from *p*(*x*, *t*): either through mortality or arrival onto the farm. Therefore, to calculate the time of arrival onto the farm, we first rescale the density of lice by their probability of survival up to time *t*, $p(x,t)=e^{-\mu t}\tilde{\;p}(x,t)$, so that e^−*μt*^ is the probability that lice have survived up to time *t* and $\tilde{\;p}(x,t)$ is the probability density of lice that are in the channel at position *x* at time *t* given that they have survived. The equation describing $\tilde{\;p}(x,t)$ is

2.8 $$\frac{\partial }{\partial t}\tilde{\;p}(x,t)=-\frac{\partial }{\partial x}(v\tilde{\;p}(x,t))+\frac{\partial^{2}}{\partial x^{2}}(D\tilde{\;p}(x,t))-h(x)\alpha \tilde{\;p}(x,t)$$

2.9 $$\tilde{\;p}(x,0)=\delta (x-x_{0})$$

2.10 $$\begin{array}{rl} \mathrm{and}\;\;h(x) & =\left\{ \begin{array}{cc} 1 & x\in [0,L]\\ 0 & \text{otherwise}, \end{array}\right.\; \end{array}$$

with the same necessary boundary conditions as before. Now that $\tilde{\;p}(x,t)$ describes the probability density of lice in the channel that have survived up to time *t*, the only way for lice to be removed from $\tilde{\;p}(x,t)$ is if they arrive on the farm, and we use this fact to calculate the arrival time.

Let *T* be the random variable describing the time of arrival onto the farm. We are interested in calculating *f*(*t*), the density of the arrival time distribution, where $\int_{0}^{t}f\;(\tau )\;d\tau =\text{Pr}(T<t)$. The integral $\int_{-\infty }^{\infty }\tilde{\;p}(x,t)\;dx$ is the probability that lice are still in the water column and have not yet arrived onto the farm, thus $\int_{-\infty }^{\infty }\tilde{\;p}(x,t)\;dx=\text{Pr}(T>t)=1-\text{Pr}(T<t)$. The arrival time distribution *f*(*t*) can, therefore, be given by

$$f(t)=-\frac{d}{dt}\int_{-\infty }^{\infty }\tilde{\;p}(x,t)\;dx.$$

If we integrate equation (2.8) in space from −∞ to ∞, then the advection and diffusion terms disappear due to the boundary conditions and we are left with

$$\frac{d}{dt}\int_{-\infty }^{\infty }\tilde{\;p}(x,t)\;dx=-\alpha \int_{0}^{L}\tilde{\;p}(x,t)\;dx.$$

Substituting this equation into the one for *f*(*t*) we find that

2.11 $$f(t)=\alpha \int_{0}^{L}\tilde{\;p}(x,t)\;dx.$$

Therefore, in order to solve *f*(*t*) we must first solve $\tilde{\;p}(x,t)$. Before turning our attention to this solution, there are a couple details which are important to note. First, because *f*(*t*) is the density of the arrival times of sea lice that arrive on the farm, $\int_{0}^{\infty }f(t)\;dt$ will only equal 1 if all lice eventually arrive onto the farm. For sea lice passing by salmon farms, the arrival rate *α* will be quite small, as farms are often located on the edge of large channels, and we are approximating the entire channel with a one-dimensional domain. Therefore, most of the lice released will not arrive onto the farm, an assumption which we will make explicit in the following section. Second, because we have removed mortality from the equation describing $\tilde{\;p}(x,t)$, *f*(*t*) is the probability density of arrival at time *t*, given that lice survive up to time *t*. The density of lice that survive up to time *t* and then arrive onto the farm is e^−*μt*^ *f*(*t*).

#### 2.2.1. Calculating arrival time via asymptotic analysis

The solution, $\tilde{\;p}(x,t)$, to equation (2.8) is difficult to solve exactly and so to find an approximate analytical solution to $\tilde{\;p}(x,t)$ and *f*(*t*) we perform an asymptotic analysis and solve the first-order solution. To find a small parameter around which to perform the asymptotic analysis we first need to non-dimensionalize our system. There are many different possibilities for non-dimensionalization, and in our case we choose to non-dimensionalize time as $\tilde{t}=D/L^{2}t$ and space as $\tilde{x}=x/L$. Using these non-dimensional parameters we can rewrite equation (2.8) as

2.12 $$\frac{\partial }{\partial \tilde{t}}\tilde{\;p}(\tilde{x},\tilde{t})=-\frac{\partial }{\partial \tilde{x}}\left(\frac{vL}{D}\tilde{\;p}(\tilde{x},\tilde{t})\right)+\frac{\partial^{2}}{\partial {\tilde{x}}^{2}}\tilde{\;p}(\tilde{x},\tilde{t})-h(\tilde{x})\frac{\alpha L^{2}}{D}\tilde{\;p}(\tilde{x},\tilde{t})$$

2.13 $$\tilde{\;p}(\tilde{x},0)=\delta \left(\tilde{x}-\frac{x_{0}}{L}\right)$$

2.14 $$\begin{array}{rl} \mathrm{and}\;\;h(\tilde{x}) & =\left\{ \begin{array}{cc} 1 & \tilde{x}\in [0,1]\\ 0 & \text{otherwise}. \end{array}\right. \end{array}$$

Along with non-dimensionalizing the equations for $\tilde{\;p}(\tilde{x},\tilde{t})$, we must also write *f*(*t*) in terms of its non-dimensional form so that it is clear how to redimensionalize *f*(*t*) to fit to data. Previously, we demonstrated that *f*(*t*) could be calculated as

$$f(t)=-\frac{d}{dt}\int_{-\infty }^{\infty }\tilde{\;p}(x,t)\;dx.$$

In terms of the new non-dimensional time and space variables, $\tilde{t}$ and $\tilde{x}$, this can be rewritten as

$$f(\tilde{t})=-\frac{D}{L}\frac{d}{d\tilde{t}}\int_{-\infty }^{\infty }\tilde{\;p}(\tilde{x},\tilde{t})\;d\tilde{x}.$$

From the formulation of $\tilde{\;p}(\tilde{x},\tilde{t})$ in equation (2.12), we can see that the non-dimensional rate of removal of $\tilde{\;p}$ will be $-d/d\tilde{t}\int_{-\infty }^{\infty }\tilde{\;p}(\tilde{x},\tilde{t})\;d\tilde{x}$, in the same manner as the dimensional removal rate, *f*(*t*), was calculated in the previous section. Therefore, if we let

$$\tilde{\;f}(\tilde{t})=-\frac{d}{d\tilde{t}}\int_{-\infty }^{\infty }\tilde{\;p}(\tilde{x},\tilde{t})\;d\tilde{x}$$

be the non-dimensional arrival time distribution, then we can write the dimensional arrival time as

2.15 $$f(\tilde{t})=\frac{D}{L}\tilde{\;f}(\tilde{t}).$$

In the non-dimensionalization process of $\tilde{\;p}(\tilde{x},\tilde{t})$ three dimensionless parameters appear: *x*_0_/*L*, *vL*/*D* and *αL*^2^/*D*. In the Broughton Archipelago, advection and diffusion have previously been estimated as *v* = 0.0645 km h^−1^ and *D* = 0.945 km^2^ h^−1^ (table 1), and the length of the average farm is around *L* = 0.1 km. To roughly estimate the magnitude of the arrival rate in one dimension, *α*, we must first make some assumptions about the arrival rate of sea lice over a salmon farm in two dimensions. Let *β* be the actual rate of arrival of lice onto a farm when they are in the water column directly over a farm. A recent laboratory-based experimental study found that 12–56% of copepodites can attach to salmon after one hour in semi-stagnant water [36]. Using a simple exponential waiting time model, *P*(*t*) = 1 − e^−*βt*^, where *P* is the probability of attachment and *β* is the attachment rate, an upper estimate of the attachment rate would be *β* = 0.821 h^−1^. To calculate *α*, we assume that the ratio of *α*/*β* = 0.012, which in physical terms means that the two-dimensional area taken up by the farm is roughly 0.012 of the area of the channel at the location of the farm. At the particular farm to which we fit the model, the farm is roughly 50 m wide and the channel is 4 km wide, so *α*/*β* ≈ 0.05/4 = 0.0125. When we fit the model we find that the estimate of *α*/*β* in fact ranges from 0.012 to 0.006 (table 2). Thus taking the values of *α*/*β* = 0.012 and *β* = 0.821 as rough maximum estimates, we assume *α* ≤ 0.01. Based on these parameter estimates and assumptions we choose *αL*^2^/*D* to be the small parameter around which we perform our asymptotic approximation.

**Table 1.** Comparison of parameter estimates in this study to others in the Broughton Archipelago.

parameter	description	analytical model	Krkosek *et al.* [23]	Brooks [31]	Krkosek *et al.* [22]	Foreman *et al.* [35]
*v*	advection	0.149 km h^−1^	—	0.0648 km h^−1^	0.0379 km h^−1^	0.2088 km h^−1^
*D*	diffusion	0.617 km^2^ h^−1^	0.945 km^2^ h^−1^	—	0.321 km^2^ h^−1^	—
*μ*_*n*_	nauplius mortality rate	0.009 h^−1^	—	—	—	—
*μ*_*c*_	copepodite mortality rate	0.012 h^−1^	0.0083 h^−1^	—	—	—

**Table 2.** Parameter estimates of best-fit models under nonlinear least squares.

parameter	description	inert (figure 4*a*)	with survival (figure 4*b*)	with survival and maturation (figure 4*c*)
*v*	advection	0.143 km h^−1^	0.175 km h^−1^	0.149 km h^−1^
*D*	diffusion	0.371 km^2^ h^−1^	0.165 km^2^ h^−1^	0.617 km^2^ h^−1^
*α*/*β*	ratio of 1D to 2D arrival rate	0.012	0.012	0.006
*μ*	combined mortality rate	—	0.020 h^−1^	—
*μ*_*n*_	nauplius mortality rate	—	—	0.009 h^−1^
*μ*_*c*_	copepodite mortality rate	—	—	0.012 h^−1^
*δ*_*m*_	median nauplius maturation time	—	—	251 h
*δ*_*s*_	maturation shape parameter	—	—	8.94
*x*_0_	position of initial farm (fixed)	−13.5 km	−13.5 km	−13.5 km
*L*	length of receiving farm (fixed)	0.1 km	0.1 km	0.1 km

Let *z* = *x*_0_/*L*, $\epsilon =\alpha L^{2}/D$ (<1.1 × 10^−4^) and *ω* = *vL*/*D* (6.83 × 10^−3^). Then we can rewrite equation (2.12) as

2.16 $$\frac{\partial }{\partial \tilde{t}}\tilde{\;p}(\tilde{x},\tilde{t})=-\frac{\partial }{\partial \tilde{x}}(\omega \tilde{\;p}(\tilde{x},\tilde{t}))+\frac{\partial^{2}}{\partial {\tilde{x}}^{2}}\tilde{\;p}(\tilde{x},\tilde{t})-\epsilon h(\tilde{x})\tilde{\;p}(\tilde{x},\tilde{t})$$

2.17 $$\tilde{\;p}(\tilde{x},0)=\delta (\tilde{x}-z)$$

2.18 $$\mathrm{and}\;\;\begin{array}{rl} h(\tilde{x}) & =\left\{ \begin{array}{cc} 1 & \tilde{x}\in [0,1]\\ 0 & \text{otherwise}. \end{array}\right. \end{array}$$

In terms of $\tilde{\;f}(\tilde{t})$, if we integrate both sides of equation (2.16) on space from −∞ to ∞, then we can write

$$\tilde{\;f}(\tilde{t})=\epsilon \int_{0}^{1}\tilde{\;p}(\tilde{x},\tilde{t})\;d\tilde{x}.$$

We assume that $\tilde{\;p}(\tilde{x},\tilde{t})$ can be expressed as a regular asymptotic expansion in epsilon,

$$\tilde{\;p}(\tilde{x},\tilde{t})={\tilde{\;p}}_{0}(\tilde{x},\tilde{t})+\epsilon {\tilde{\;p}}_{1}(\tilde{x},\tilde{t})+O(\epsilon^{2})$$

and then can express $\tilde{\;f}(\tilde{t})$ as

$$\tilde{\;f}(\tilde{t})=\epsilon \int_{0}^{1}{\tilde{\;p}}_{0}(\tilde{x},\tilde{t})\;d\tilde{x}+O(\epsilon^{2}).$$

Substituting the expansion for $\tilde{\;p}(\tilde{x},\tilde{t})$ into equation (2.16) and matching terms of order $\epsilon^{0}$, we have

2.19 $$\frac{\partial }{\partial \tilde{t}}{\tilde{\;p}}_{0}(\tilde{x},\tilde{t})=-\frac{\partial }{\partial \tilde{x}}(\omega {\tilde{\;p}}_{0}(\tilde{x},\tilde{t}))+\frac{\partial^{2}}{\partial {\tilde{x}}^{2}}{\tilde{\;p}}_{0}(\tilde{x},\tilde{t})$$

and

2.20 $${\tilde{\;p}}_{0}(\tilde{x},0)=\delta (\tilde{x}-z),$$

which has the solution

2.21 $${\tilde{\;p}}_{0}(\tilde{x},\tilde{t})=\frac{1}{\sqrt{4\pi \tilde{t}}}\;e^{-{\left(\tilde{x}-z-\omega \tilde{t}\right)}^{2}/4\tilde{t}}.$$

Therefore, $\tilde{\;f}(\tilde{t})$, up to order $\epsilon^{2}$, is given by

2.22 $$\;\tilde{\;f}(\tilde{t})=\epsilon \int_{0}^{1}p_{0}(\tilde{x},\tilde{t})\;d\tilde{x}+O(\epsilon^{2})$$

2.23 $$=\epsilon \int_{0}^{1}\frac{1}{\sqrt{4\pi \tilde{t}}}\;e^{-{\left(\tilde{x}-z-\omega \tilde{t}\right)}^{2}/4\tilde{t}}\;d\tilde{x}+O(\epsilon^{2}).$$

Returning to our original dimensional parameters, the dimensional form of the arrival time distribution is

2.24 $$\;f(t)=\alpha \int_{0}^{L}\frac{1}{\sqrt{4\pi Dt}}\;e^{-{\left(x-x_{0}-vt\right)}^{2}/4Dt}\;dx$$

2.25 $$=\frac{\alpha }{2}\left(\text{erf}\left(\frac{x_{0}+vt}{\sqrt{4Dt}}\right)-\text{erf}\left(\frac{x_{0}+vt-L}{\sqrt{4Dt}}\right)\right).$$

#### 2.2.2. Including survival and maturation

In the previous section, we ignored the fact that sea lice larvae actually have two developmental stages: a non-infectious nauplius stage and an infectious copepodite stage. In the nauplius stage, sea lice larvae cannot attach to salmonid hosts even if they come in close contact; it is only in the copepodite stage that sea lice are able to attach to hosts. To capture the difference in host attachment between these two stages, we model the densities of the nauplius (*p*_*n*_(*x*, *t*)) and copepodite (*p*_*c*_(*x*, *t*)) stages at position x∈R and time *t* ≥ 0 with the following differential equations:

2.26 $$\frac{\partial }{\partial t}p_{n}(x,t)=-\underset{\mathrm{advection}}{\underbrace{\frac{\partial }{\partial x}(vp_{n}(x,t))}}+\underset{\mathrm{diffusion}}{\underbrace{\frac{\partial^{2}}{\partial x^{2}}(Dp_{n}(x,t))}}-\underset{\mathrm{nauplius}\;\mathrm{mortality}}{\underbrace{\mu_{n}p_{n}(x,t)}}-\underset{\mathrm{maturation}}{\underbrace{m(t)p_{n}(x,t)}}$$

2.27 $$p_{n}(x,0)=\delta (x-x_{0})$$

2.28 $$\;\begin{array}{rl} \frac{\partial }{\partial t}p_{c}(x,t) & =\underset{\mathrm{maturation}}{\underbrace{m(t)p_{n}(x,t)}}-\underset{\mathrm{advection}}{\underbrace{\frac{\partial }{\partial x}(vp_{c}(x,t))}}+\underset{\mathrm{diffusion}}{\underbrace{\frac{\partial^{2}}{\partial x^{2}}(Dp_{c}(x,t))}}\\ & \;-\underset{\mathrm{copepodite}\;\mathrm{mortality}}{\underbrace{\mu_{c}p_{c}(x,t)}}-\underset{\mathrm{arrival}\;\mathrm{onto}\;\mathrm{farm}}{\underbrace{\alpha h(x)p_{c}(x,t)}} \end{array}$$

2.29 $$p_{c}(x,0)=0$$

2.30 $$\begin{array}{rl} \mathrm{and}\;\;h(x) & =\left\{ \begin{array}{cc} 1 & x\in [0,L]\\ 0 & \text{otherwise}, \end{array}\right.\; \end{array}$$

where *μ*_*i*_ is the mortality rate in stage *i* and *m*(*t*) is the maturation rate of nauplii to copepodites. Note that all sea lice hatch as nauplii.

Now it is only infectious copepodites that can arrive onto the receiving farm, and so in order to calculate the arrival time of larvae we need to calculate the rate of removal of infectious larvae from the water column. However, we do not want to count infectious larvae that die, so first we need to separate out mortality from the two equations. Let $p_{c}(x,t)=e^{-\mu_{c}t}{\tilde{\;p}}_{c}(x,t)$ and $p_{n}(x,t)=e^{-\mu_{n}t}{\tilde{\;p}}_{n}(x,t)$, where $e^{-\mu_{c}t}$ and $e^{-\mu_{n}t}$ are the probabilities that lice survive up to time *t* in the copepodite and nauplius stages, respectively. In the previous section, where there was only one stage, the arrival time distribution was given by $f(t)=-d/dt\int_{-\infty }^{\infty }\tilde{\;p}(x,t)\;dx$, but now that there are two stages with different death rates the arrival time distribution will be given by

2.31 $$e^{-\mu_{c}t}f(t)=-e^{\mu_{c}t}\frac{d}{dt}\int_{-\infty }^{\infty }{\tilde{\;p}}_{c}(x,t)\;dx-e^{-\mu_{n}t}\frac{d}{dt}\int_{-\infty }^{\infty }{\tilde{\;p}}_{n}(x,t)\;dx.$$

To calculate the arrival time we can rewrite equations (2.26)–(2.30) as

2.32 $$\begin{array}{rl} e^{-\mu_{n}t}\frac{\partial }{\partial t}{\tilde{\;p}}_{n}(x,t) & =-e^{-\mu_{n}t}\frac{\partial }{\partial x}(v{\tilde{\;p}}_{n}(x,t))+e^{-\mu_{n}t}\frac{\partial^{2}}{\partial x^{2}}(D{\tilde{\;p}}_{n}(x,t))\\ & \;-e^{-\mu_{n}t}m(t){\tilde{\;p}}_{n}(x,t) \end{array}$$

2.33 $${\tilde{\;p}}_{n}(x,0)=\delta (x-x_{0})$$

2.34 $$\begin{array}{rl} e^{-\mu_{c}t}\frac{\partial }{\partial t}{\tilde{\;p}}_{c}(x,t) & =e^{-\mu_{n}t}m(t){\tilde{\;p}}_{n}(x,t)-e^{-\mu_{c}t}\frac{\partial }{\partial x}(v{\tilde{\;p}}_{c}(x,t))\\ & \;+e^{-\mu_{c}t}\frac{\partial^{2}}{\partial x^{2}}(D{\tilde{\;p}}_{c}(x,t))-e^{-\mu_{c}t}\alpha h(x){\tilde{\;p}}_{c}(x,t) \end{array}$$

2.35 $${\tilde{\;p}}_{c}(x,0)=0$$

2.36 $$\begin{array}{rl} \mathrm{and}\;\;h(x) & =\left\{ \begin{array}{cc} 1 & x\in [0,L]\\ 0 & \text{otherwise}. \end{array}\right. \end{array}$$

Then substituting equations (2.32) and (2.34) into equation (2.31), we find that

$$f_{c}(t)=\alpha \int_{0}^{L}{\tilde{\;p}}_{c}(x,t)\;dx,$$

where we use the subscript *c* to denote the arrival time of lice which have matured into copepodites.

Once again, to calculate the arrival time density we will need to solve the equations governing the louse distribution in the channel using an asymptotic analysis, this time with the addition of Green’s functions.

First, let us formulate the copepodid density, ${\tilde{\;p}}_{c}(x,t)$, in terms of a Green’s function. The Green’s function describes the movement of copepodites, as described by equation (2.34), but without the source of maturing nauplii. The Green’s function is then convolved with the source function: the maturing nauplii which are entering the copepodid stage. The copepodid density can then be written as

2.37 $${\tilde{\;p}}_{c}(x,t)=\int_{0}^{t}\int_{-\infty }^{\infty }G(x-\xi ,t-\tau )s(\xi ,\tau )\;d\xi \;d\tau ,$$

where $s(\xi ,\tau )=e^{(\mu_{c}-\mu_{n})\tau }m(\tau ){\tilde{\;p}}_{n}(\xi ,\tau )$ and *G*(*x*, *t*) solves

2.38 $$\frac{\partial }{\partial t}G(x,t)=-\frac{\partial }{\partial x}(vG(x,t))+\frac{\partial^{2}}{\partial x^{2}}(DG(x,t))-\alpha h(x)G(x,t)$$

2.39 G(x,0)=δ(x)

2.40 $$\begin{array}{rl} \mathrm{and}\;\;h(x) & =\left\{ \begin{array}{cc} 1 & x\in [0,L]\\ 0 & \text{otherwise}. \end{array}\right. \end{array}$$

Similar to before, the equation governing *G*(*x*, *t*) is difficult to solve directly, and so we non-dimensionalize the equations and then perform an asymptotic analysis in a small parameter. We non-dimensionalize equations (2.38)–(2.40) and non-dimensionalize the formula for ${\tilde{\;p}}_{c}(x,t)$ (equation (2.37)) directly. As before, let $\tilde{t}=(D/L^{2})t$ and $\tilde{x}=x/L$. Then the nauplius and copepodid system can be reformulated in a non-dimensional form as

2.41 $$\frac{\partial }{\partial \tilde{t}}{\tilde{\;p}}_{n}(\tilde{x},\tilde{t})=-\frac{\partial }{\partial \tilde{x}}\left(\frac{vL}{D}{\tilde{\;p}}_{n}(\tilde{x},t)\right)+\frac{\partial^{2}}{\partial {\tilde{x}}^{2}}{\tilde{\;p}}_{n}(\tilde{x},\tilde{t})-\frac{L^{2}}{D}m(\tilde{t})p_{n}(\tilde{x},\tilde{t})$$

2.42 $${\tilde{\;p}}_{n}(\tilde{x},0)=\delta \left(\tilde{x}-\frac{x_{0}}{L}\right)$$

2.43 $$\frac{\partial }{\partial \tilde{t}}G(\tilde{x},\tilde{t})=-\frac{\partial }{\partial \tilde{x}}\left(\frac{vL}{D}G(\tilde{x},\tilde{t})\right)+\frac{\partial^{2}}{\partial {\tilde{x}}^{2}}G(\tilde{x},\tilde{t})-\frac{\alpha L^{2}}{D}h(x)G(x,t)$$

2.44 $$G(\tilde{x},0)=\delta (\tilde{x})$$

2.45 $$\begin{array}{rl} h(x) & =\left\{ \begin{array}{cc} 1 & x\in [0,L]\\ 0 & \text{otherwise} \end{array}\right. \end{array}$$

2.46 $$\begin{array}{rl} \mathrm{and}\;\;{\tilde{\;p}}_{c}(\tilde{x},\tilde{t}) & =\frac{L^{3}}{D}\int_{0}^{\tilde{t}}\int_{-\infty }^{\infty }G(\tilde{x}-\tilde{\xi },\tilde{t}-\tilde{\tau })\times e^{\left(\mu_{c}-\mu_{n})(L^{2}\tilde{\tau }/D\right)}m(\tilde{\tau }){\tilde{\;p}}_{n}(\tilde{\xi },\tilde{\tau })\;d\tilde{\xi }\;d\tilde{\tau }. \end{array}$$

Similar to the previous section, we also need to write *f*(*t*) in terms of the new non-dimensional space and time variables. Rescaling equation (2.31) we have

2.47 $$\begin{array}{rl} e^{-\mu_{c}(L^{2}\tilde{t}/D)}f_{c}(\tilde{t}) & =\frac{D}{L}(-e^{\mu_{c}(L^{2}\tilde{t}/D)}\frac{d}{d\tilde{t}}\int_{-\infty }^{\infty }{\tilde{\;p}}_{c}(\tilde{x},\tilde{t})\;d\tilde{x}\\ & \;-e^{-\mu_{n}(L^{2}\tilde{t}/D)}\frac{d}{d\tilde{t}}\int_{-\infty }^{\infty }{\tilde{\;p}}_{n}(\tilde{x},\tilde{t})\;d\tilde{x}), \end{array}$$

so if we let the non-dimensional version of our arrival time distribution be given by

2.48 $$\begin{array}{rl} e^{-\mu_{c}(L^{2}\tilde{t}/D)}{\tilde{\;f}}_{c}(\tilde{t}) & =\left(-e^{\mu_{c}(L^{2}\tilde{t}/D)}\frac{d}{d\tilde{t}}\int_{-\infty }^{\infty }{\tilde{\;p}}_{c}(\tilde{x},\tilde{t})\;d\tilde{x}-e^{-\mu_{n}(L^{2}\tilde{t}/D)}\frac{d}{d\tilde{t}}\int_{-\infty }^{\infty }{\tilde{\;p}}_{n}(\tilde{x},\tilde{t})\;d\tilde{x}\right) \end{array}$$

then the relationship between the dimensional and non-dimensional forms of the arrival time is

$$f_{c}(\tilde{t})=\frac{D}{L}{\tilde{\;f}}_{c}(\tilde{t}).$$

For our small parameter, we again choose $\epsilon =\alpha L^{2}/D$, around which we perform our expansion, and *ω* = *vL*/*D* as the other non-dimensional parameter. We can then expand $G(\tilde{x},\tilde{t})=G_{0}(\tilde{x},\tilde{t})+\epsilon G_{1}(\tilde{x},\tilde{t})+O(\epsilon^{2})$, along with the corresponding ${\tilde{\;p}}_{c}(\tilde{x},\tilde{t})={\tilde{\;p}}_{c0}(\tilde{x},\tilde{t})+\epsilon {\tilde{\;p}}_{c1}(\tilde{x},\tilde{t})+O(\epsilon^{2})$.

Then using these new parameters and adding together the terms on the right-hand side of equation (2.48), the non-dimensional version of the arrival time distribution, $\tilde{\;f}(\tilde{t})$, is

2.49 $${\tilde{\;f}}_{c}(\tilde{t})=\epsilon \int_{0}^{1}{\tilde{\;p}}_{c0}(\tilde{x},\tilde{t})\;d\tilde{x}+O(\epsilon^{2}).$$

Therefore, to find ${\tilde{\;f}}_{c}(\tilde{t})$ we need to calculate $G_{0}(\tilde{x},\tilde{t})$ and ${\tilde{\;p}}_{c0}(\tilde{x},\tilde{t})$. Focusing first on $G(\tilde{x},\tilde{t})$ we can match terms of order $\epsilon^{0}$ in equation (2.43) to arrive at

2.50 $$\frac{\partial }{\partial \tilde{t}}G_{0}(\tilde{x},\tilde{t})=-\frac{\partial }{\partial \tilde{x}}(\omega G_{0}(\tilde{x},\tilde{t}))+\frac{\partial^{2}}{\partial {\tilde{x}}^{2}}G_{0}(\tilde{x},\tilde{t})$$

and

2.51 $$G_{0}(\tilde{x},0)=\delta (\tilde{x})$$

which has the solution

2.52 $$G_{0}(\tilde{x},\tilde{t})=\frac{1}{\sqrt{4\pi \tilde{t}}}\;e^{-{\left(\tilde{x}-\omega \tilde{t}\right)}^{2}/4\tilde{t}}$$

with the corresponding solution in ${\tilde{\;p}}_{c0}(\tilde{x},\tilde{t})$:

2.53 $${\tilde{\;p}}_{c0}(\tilde{x},\tilde{t})=\frac{L^{3}}{D}\int_{0}^{\tilde{t}}\int_{-\infty }^{\infty }G_{0}(\tilde{x}-\tilde{\xi },\tilde{t}-\tilde{\tau })\;e^{\left(\mu_{c}-\mu_{n})(L^{2}\tilde{\tau }/D\right)}m(\tilde{\tau }){\tilde{\;p}}_{n}(\tilde{\xi },\tilde{\tau })\;d\tilde{\xi }\;d\tilde{\tau }.$$

Therefore, the arrival time distribution, up to order $\epsilon^{2}$, is given by ${\tilde{\;f}}_{c}(\tilde{t})=$ $\epsilon \int_{0}^{1}{\tilde{\;p}}_{c0}(\tilde{x},\tilde{t})\;d\tilde{x}+O(\epsilon^{2})$ along with equations (2.52) and (2.53). Before writing out ${\tilde{\;f}}_{c}(\tilde{t})$ explicitly, we will first redimensionalize the arrival time distribution, as this is what will be fitted to data. In its dimensional form with the original time and space variables we have

2.54 $$f_{c}(t)\approx \alpha \int_{0}^{L}\int_{0}^{t}\int_{-\infty }^{\infty }G_{0}(x-\xi ,t-\tau )\;e^{(\mu_{c}-\mu_{n})\tau }m(\tau ){\tilde{\;p}}_{n}(\xi ,\tau )\;d\xi \;d\tau$$

2.55 $$G_{0}(x,t)=\frac{1}{\sqrt{4\pi Dt}}\;e^{-{\left(x-vt\right)}^{2}/4Dt}$$

2.56 $$\mathrm{and}\;\;{\tilde{\;p}}_{n}(x,t)=\frac{1}{\sqrt{4\pi Dt}}\;e^{-{\left(x-x_{0}-vt\right)}^{2}/4Dt}\;e^{-\int_{0}^{t}m(u)\;du}.$$

### 2.3. Coupled biological–physical particle tracking simulation

Now that we have an analytical solution for the arrival time distribution we turn to measuring the arrival time directly for sea lice dispersing between farms in the Broughton Archipelago. To do so we use a bio-physical particle tracking simulation. This particle tracking simulation uses an underlying ocean circulation model, the FVCOM [37]. The simulation period of the FVCOM was between 1 March and 31 July 2009, to coincide with the outmigration of juvenile pink and chum salmon in that year. More details on the FVCOM simulation can be found in [10,35], but briefly the FVCOM uses data on tides, wind, surface heating and river discharge from the six major rivers in the Broughton as input to simulate three-dimensional ocean velocity, temperature and salinity. The FVCOM uses an unstructured grid to solve the necessary hydrodynamic equations, which allows for a more realistic simulation of ocean circulation near the complex coastlines of the Broughton Archipelago. The FVCOM currents arising from this 2009 simulation were compared with observations from 12 current meter moorings and found to be in relatively close agreement [35].

Hourly output from the FVCOM was used as input into an offline bio-physical particle tracking simulation, details of which can be found in [10]. The physical component of the particle tracking simulation determines how sea lice particles move based on the current that they experience from the output of the FVCOM, and the biological component determines how they survive and mature based on the local salinity and temperature that they experience. Particles are first released from farms as pre-infectious nauplii and then mature into infectious copepodites. The development time from nauplii to copepodites is based on the temperature (*T*) that particles experience, and is given by the simplified Bělehrádek function [38]:

2.57 $$\tau (T)={\left[\frac{\beta_{1}}{T-10+\beta_{1}\beta_{2}}\right]}^{2},$$

where *β*_1_ = 24.79 and *β*_2_ = 0.525. With this parametrization, *β*_1_ is a shape parameter and $\beta_{2}^{-2}$ is the average time to development at 10°C. As a particle will experience different temperatures over its lifetime, to track maturity each particle is given a maturity value (*M*). The maturity value starts at 0 for a newly released nauplius and then updates via

2.58 $$M_{t}=M_{t-1}+\frac{\Delta t}{\tau (T)}.$$

Once the maturity value, *M*, reaches 1, the particle moults into a copepodite.

The survival probability of each particle is given by

$$S(t)=e^{-\mu t},$$

where the survival coefficient, *μ*, is constant at 0.31 per day when the salinity is above 30 ppt for the nauplius stage, and at less than 30 ppt is given by

2.59 μ=5.11−0.16×salinity.

Once the particles mature into copepodites (with a maturity coefficient greater than or equal to 1), then the survival coefficient is constant at 0.22 per day. The constant survival in the mature copepodite stage is due to a lack of consensus among studies on how copepodite survival changes with temperature and salinity.

In the bio-physical particle tracking simulation, sea lice particles do not perform any diel vertical migration. While there are laboratory studies that find sea lice larvae perform diel vertical migration [16], field studies in the Broughton Archipelago found that copepodites did not exhibit any depth preference during the day or nighttime [15]. However, there is evidence that sea lice larvae can use their limited swimming abilities to remain in the top few metres of the water column [39] and so sea lice particles in the simulation were constrained the remain in the top 5 m.

To determine the trajectories of sea lice originating from farms in the Broughton Archipelago, 50 particles were released per hour from each of the 20 active farms (during 2009) and tracked for 11 days using the offline particle tracking model. Sea lice larvae (nauplii and copepodites) are non-feeding and so it is assumed that if they have not yet found a host after 11 days the sea lice larvae either die or are no longer capable of attaching to the host due to a lack of energy reserves [15,40]. The first day of release was 14 March, and the last day of release was 20 July 2009. In this paper, to fit the analytical arrival time distribution, we use the particles released on 2 May 2009 (CRD 50 in [10]). We fitted to data from this day as it was the one chosen as being representative of an ‘average’ day in [10]. However, we also present the results of a fit to a high connectivity day, 11 May 2009 (CRD 59 in [10]), in the electronic supplementary material.

The 24 h of particle releases (24 releases × 50 particles per release) on 2 May 2009 were combined into one cohort so that the time of release of the entire cohort begins at *t* = 0. The amalgamation of 24 h of releases on one day is to smooth out the effect that the daily tidal cycle may have on any given individual release. When fitting arrival time using the amalgamation of 24 h of particle releases, the constant advection coefficient in the model captures directional water movement due to river runoff and the diffusion in the model coefficient captures the average mixing due to tidal flow and wind currents.

For the cohort released on 2 May, we created kernel density estimates (KDEs) using the particle locations at every hour over the 11 days that they were tracked, for a total of 265 KDEs. The idea behind kernel density estimation is to create a distribution from individual particle locations by applying a smoothed Gaussian kernel around each particle location and then adding each kernel to create a distribution. An alternative to using KDEs would be to simply count the number of particles over the receiving farm at every hour. However in our case, due to the relatively small number of particles released, out of the 264 h for which the particles were tracked there were only 28 h in which there was one or two particles over the location of the receiving farm. Kernel density estimation solves this problem by creating smooth densities from the discrete particle locations, so that particles very near to but not directly over the farm contribute to a higher density of lice over the farm.

In order to create the KDEs, a bandwidth must be chosen which controls the variance of the Gaussian kernel created around each particle. Here, we use a bandwidth of 1/8 of the minimum extent of the *x* or *y* coordinates of the particles at each hour, so that as the particles spread out the bandwidth will increase. This bandwidth was chosen based on work by Cantrell *et al.* [10,41] who used the same bandwidth to fit KDEs to sea lice particles in the Broughton Archipelago. A sensitivity analysis of this choice showed that parameter values did not differ dramatically given other choices of bandwidth. This sensitivity analysis, along with KDE plots for the bandwidth of 1/8 at certain time intervals, is available in the electronic supplementary material. The rest of the specific details behind the kernel density estimation process for the sea lice particles can be found in [10].

### 2.4. Model fitting

In order to use the KDEs to fit our analytical model of arrival time (2.54)–(2.56) we first need to derive an expression for arrival time based on the KDEs. The initial step in this process is to determine which farm to set as the release farm for sea lice and which to use as the receiving farm. In the Broughton Archipelago the use of a one-dimensional advection diffusion model to determine the distribution of sea lice on wild salmon from source farms has been fitted to data mainly in Tribune Channel and so to compare parameter estimates we choose farms also in Tribune. Our release farm is Glacier Falls, located in the centre of Tribune Channel and our receiving farm is Burdwood, which lies at the opening of Tribune (see figure 2).

The KDE represents the distribution of particles over space and has units of particles per kilometre squared. In order to convert this density distribution into a probability distribution, we first must divide the KDE by the total number of particles released, in this case 1200 (50 particles × 24 releases (1 per hour)). Then the new KDE represents the two-dimensional probability distribution of particles, with the integral over the entire domain equal to one; this is now the two-dimensional equivalent of *p*(*x*, *t*), which we denote *p*_*h*_(**x**, *t*).

Recall that in the one-dimensional mechanistic model, the density of the arrival time was calculated as

$$f(t)=\alpha \int_{0}^{L}p(x,t)\;dx$$

and so to calculate the arrival time for the particle tracking simulations we take the value of the rescaled KDE at the position of the receiving farm and multiply it by the area of the raster cell (approximately the size of the farm), which is 0.01 km^2^. The only unknown quantity is the rate of arrival of lice onto the farm over which they are passing, which we call *β* (this is the two-dimensional equivalent of *α*). As detailed at the start of §2.2.1, an upper estimate of the arrival rate is *β* = 0.821 h^−1^. We assume here that this rate is small enough such that the number of lice that arrive onto the farm is small compared to the total number of lice in the rest of the domain, and thus we do not discount future KDEs by any proportion of lice that have potentially arrived on a farm.

The hydrodynamic equivalent of the arrival time distribution can then be calculated as

$$f_{h}(t)=\beta \int_{\mathrm{farm}}p_{h}(x,t)\;dA,$$

where the subscript *h* refers to the fact that *f*_*h*_(*t*) and *p*_*h*_(**x**, *t*) are approximations from the hydrodynamic model. We want to fit *f*_*h*_(*t*) to our original arrival time distribution $f(t)=\alpha \int_{0}^{L}p(x,t)\;dx$. We could use our assumption of the arrival rate for the hydrodynamic model, *β*, to fit the arrival rate of the one-dimensional model *α*; however, due to the uncertainties in *β* we instead fit

$$\left(\frac{\alpha }{\beta }\right)\int_{0}^{L}p(x,t)\;dx$$

to $\int_{\mathrm{farm}}p_{h}(x,t)\;dA$, calculated from kernel density estimation. For simplicity we use *β* = 1 rather than *β* = 0.821 to calculate the arrival time in figures 4 and 5.

We fit three different mechanistic models to the arrival time from the KDEs: arrival time of inert particles, arrival time of particles which have survived and arrival time of sea lice particles that have survived and matured to their infective stage. These three models each use slightly different KDEs. For the inert particles, the KDEs are constructed solely from the positions of the sea lice particles at each time step. For the arrival time of only the particles that have survived up to *t*, the KDEs are constructed by weighting each particle by its individual survival coefficient, which depends on the local salinity that it has experienced, as described in the previous section. Details on this weighting can be found in [10]. Lastly, for the arrival time of sea lice particles that have both survived up to time *t* and matured into infectious we follow Cantrell *et al.* [10] and construct KDEs using only particles that have a maturity value greater than or equal to 0.8, and then these particles are again weighted by their survival coefficient during the construction of the KDEs. Cantrell *et al.* [10] found that the maturity model (equation (2.58)) may be too sensitive to decreases in temperature and thus setting the maturity threshold to 0.8 can mitigate the potential for over-sensitivity to low temperature. If a maturity threshold of 1 is used, the development time from nauplius to copepodite will increase.

We use nonlinear least squares to fit our arrival time models to the arrival time from the KDEs, with the size of the farm and distance of release farm fixed at *L* = 0.1 km and *x*_0_ = −13.5 km, respectively. Maximum and minimum advection speeds were visually estimated using the hourly KDEs and then the estimates were used to bound the advection parameter *v* during the nonlinear least squares. The mortality rates (*μ*, *μ*_*n*_, *μ*_*c*_) were constrained to lie within the maximum and minimum possible rates in the particle tracking model, and the ratio of the one-dimensional to two-dimensional arrival rates *α*/*β* was constrained to be less than 0.0125, which is roughly the ratio of the width of the farm to the width of the channel. The best-fit parameter estimates for all parameters can be found in table 2.

**Figure 3.**
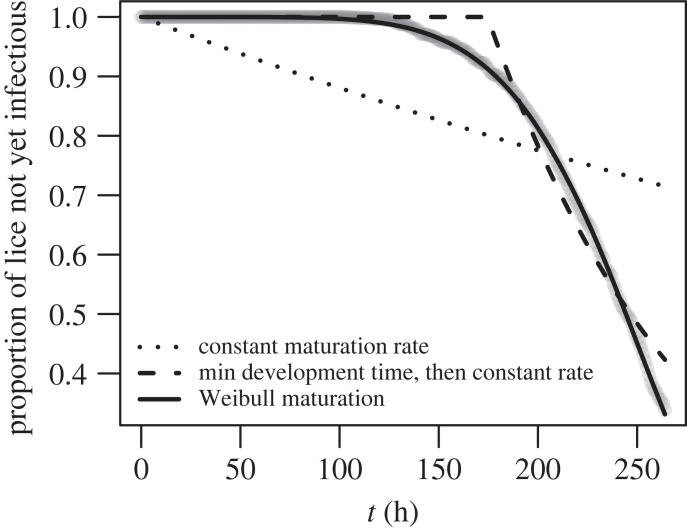
The proportion of larvae that have not yet reached a maturation level of 0.8 in the hydrodynamic model, with the best-fit lines for three different maturation functions. The dotted line is the maturation function corresponding to a constant maturation rate (e^−*mt*^), the dashed line is the maturation function for a minimum development time followed by a constant maturation rate $\left(1-H(t-t_{\min})(1-e^{-m(t-t_{\min})}\right)$, and the solid line is the Weibull maturation function ($e^{-\log(2){\left(t/\delta_{m}\right)}^{\delta_{s}}}$).

In the arrival time model that includes maturation, it is necessary to specify a maturation function in order to fit the model to the KDE data. In the sea lice literature there are several models of maturation that are used: a constant maturation rate [23,24], a strict minimum development time followed by a constant maturation rate [11,42] or a Weibull maturation function [27]. We choose to use the Weibull maturation function to model sea lice maturation, as it gave a better fit to our maturation data (figure 3) than the aforementioned alternatives. The Weibull distribution is a two parameter distribution and can be parametrized in a number of ways. We choose to follow Aldrin *et al.* [27] and use the median time to development, *δ*_*m*_, and a shape parameter, *δ*_*s*_, to define the distribution. Using these parameters the maturation rate (often called the hazard rate in survival analysis) is

2.60 $$m(t)=\log(2)\delta_{s}\delta_{m}^{-\delta_{s}}t^{\delta_{s}-1}.$$

## 3. Results

### 3.1. Arrival time of inert particles

First, we present the arrival time distribution of inert sea lice particles, which do not have any survival or maturation characteristics associated with them, but are still confined to the top 5 m of the water column. The formula for the arrival time distribution from the analytical model, given in §2.2, is

$$f(t)=\alpha \int_{0}^{L}\frac{1}{\sqrt{4\pi Dt}}\;e^{-{\left(x-x_{0}-vt\right)}^{2}/4Dt}\;dx.$$

**Figure 4.**
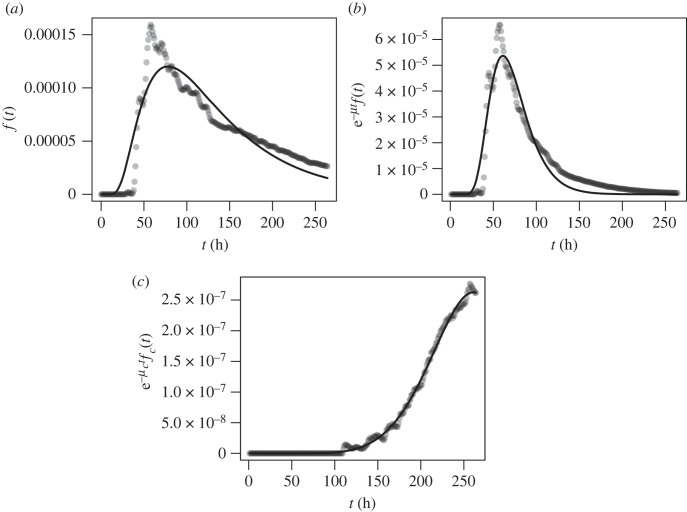
Arrival time distribution of (*a*) inert particles, (*b*) particles with survival included and (*c*) infectious copepodites. In (*a*), the points are the arrival time densities calculated from the KDEs of the particle locations, in (*b*), the points are the arrival time densities calculated from the KDEs of particle locations weighted by survival and in (*c*), the points are the arrival time densities calculated from infectious particle locations weighted by survival. The curves are the best-fit lines of the corresponding analytical models fitted to these points via nonlinear least squares. Parameter estimates for the model are given in table 2, with *β* = 1.

The fit of this distribution to the output from the hydrodynamic simulation can be seen in figure 4*a*, along with the best-fit parameter estimates in table 2.

### 3.2. Arrival time including survival

Next, we present the fit of the arrival time distribution of particles that have survived to KDEs that are weighted by survival, as described in §2.3. Therefore, the distribution we are now fitting is

$$e^{-\mu t}f(t)=\alpha \;e^{-\mu t}\int_{0}^{L}\frac{1}{\sqrt{4\pi Dt}}\;e^{-{\left(x-x_{0}-vt\right)}^{2}/4Dt}\;dx.$$

The survival function, e^−*μt*^, is the probability that a sea louse has survived up to time *t*, and $\int_{t-\epsilon }^{t+\epsilon }f(\tau )\;d\tau$ is the probability that a sea louse arrives on the second farm between t−ϵ and t+ϵ, given that it has survived. The fit of e^−*μt*^*f*(*t*) to the hydrodynamic model is shown in figure 4*b*.

### 3.3. Arrival time of sea lice (maturation and survival)

Lastly, we present the fit of the arrival time distribution of infectious sea lice particles. In the hydrodynamic simulation these particles each mature and have a survival probability based on the local salinity and temperature that they experience over their lifetime. For the one-dimensional mechanistic model, we are fitting the arrival time distribution of copepodites, *f*(*t*), offset by the probability that a copepodite survives up to time *t*, $e^{-\mu_{c}t}$. In short, we are fitting

$$e^{-\mu_{c}t}f_{c}(t)=\alpha \int_{0}^{L}\int_{0}^{t}\int_{-\infty }^{\infty }G_{0}(x-\xi ,t-\tau )\;e^{-\mu_{c}(t-\tau )}\;e^{-\mu_{n}\tau }m(\tau ){\tilde{\;p}}_{n}(\xi ,\tau )\;d\xi \;d\tau ,$$

where *G*_0_(*x*, *t*), ${\tilde{\;p}}_{n}(x,t)$ and *m*(*t*) are given by equations (2.55), (2.56) and (2.60), respectively, to the KDEs of sea lice particles that have survived and have matured from nauplii to infectious copepodites. The fit of $e^{-\mu_{c}t}f_{c}(t)$ is shown in figure 4*c* and the best-fit parameters are in table 2. The cross-infection between farms is given by

$$\int_{0}^{\infty }\;e^{-\mu_{c}t}f_{c}(t)\;dt.$$

### 3.4. Applications

Now that we have fitted our arrival time model to the hydrodynamic simulation, we aim to answer the questions posed in the Introduction surrounding the placement of salmon farms in a channel. We have a model for the distribution of arrival times of sea lice coming from a second farm, and so there are a variety of analyses that can be done with such a distribution. In this section, we will focus on how various factors affect cross-infection, or overall probability that sea lice travel from one farm to the other, given by $\int_{0}^{264}\;e^{-\mu_{c}t}f_{c}(t)\;dt$. Here, we take the integral from 0 to 264 h (11 days), as it is assumed in the particle tracking model that after 11 days sea lice larvae either die or can no longer attach to a host. For our analyses we will use our full arrival time model that includes both maturation and survival of sea lice, as we focus on how different parameters affect the total probability that lice arrive on the farm, but for other questions relating to how the advection and diffusivity affect the mean arrival time of particles, the simpler models may be more suitable.

To answer the questions posed in the Introduction, we explore how three different interactions of parameters affect the cross-infection: we vary advection and initial farm placement (*x*_0_) to answer question (ii), we vary advection (*v*) and diffusion (*D*) to answer question (iii) and we vary median maturation time (*δ*_*m*_) and initial farm placement to answer question (iv). Each of these interactions reveals a glimpse into the different factors affecting cross-infection, and together they help answer the first question posed in the Introduction. Apart from the parameters that are varying, all others will be held constant at their best-fit estimates from the nonlinear least squares fit, shown in table 2. We begin by answering the last three questions before turning our attention to the first.

#### 3.4.1. Can intermediate farm spacing maximize cross-infection?

**Figure 5.**
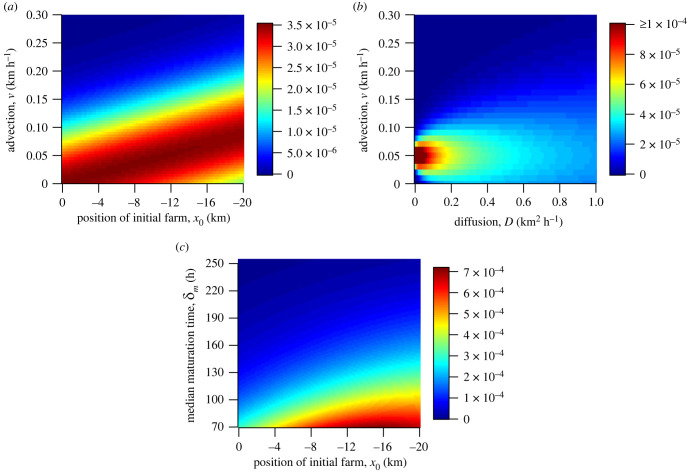
The cross-infection between farms, given by $\int_{0}^{264}\;e^{-\mu_{c}t}f(t)\;dt$, as different parameters vary: (*a*) advection and initial farm placement, (*b*) advection and diffusion and (*c*) initial farm placement and median development time. Apart from the parameters that are varying, all other parameters are held constant at their best-fit estimates shown in table 2, with *β* = 1.

By examining the effect of varying the advection coefficient and the placement of the first farm, we can see from figure 5*a* there are indeed scenarios where an intermediate spacing leads to the highest level of cross-infection. At even small advection coefficients, for example *v* = 0.05, cross-infection is maximized when the second farm is placed around 14 km away from the first. However, even if transmission to a farm 1 km away may be lower than transmission to a farm 14 km away, sea louse outbreaks on certain farms in the Broughton have been shown to be primarily driven by self-infection [43], so it is likely that local currents allow sea lice to directly mature in a farm leading to high rates of within farm infection.

In fact, the probability of arrival is maximized along the line *v* = *mx*_0_ + *b*, for some slope *m* and intercept *b*, and the probability decreases symmetrically as the intercept *b* moves away from the intercept at which the probability is maximized. Intuitively this seems to be due to the relationship between the spatial mean of the solution to equation (2.1). The solution is

$$p(x,t)=\frac{1}{\sqrt{4\pi Dt}}\;e^{-\mu t-{\left(x-x_{0}-vt\right)}^{2}/4Dt},$$

which has the spatial mean *x*_0_ + *vt*. So for a fixed maturation time, the probability of arrival should be maximized if most lice have matured before the mean density of lice moving through the channel passes by the second farm.

#### 3.4.2. How does cross-infection differ between advection versus diffusion dominated systems?

The relationship between the advection and diffusion of the system and the arrival probability is shown in figure 5*b*. We can see that for any diffusion coefficient, there is a single advection coefficient that maximizes the probability of arrival. At this maximizing advection coefficient, increasing the diffusion coefficient simply reduces the probability of arrival. Now this is in the context of a fixed release location and median maturation time, but for these fixed parameters the advection coefficient plays a large role in determining whether lice will arrive at all, and the value of the diffusion coefficient determines how large the probability of arrival will be.

#### 3.4.3. How does maturation time affect cross-infection?

The relationship between the median maturation time, *δ*_*m*_, the placement of the release farm, *x*_0_, and the arrival probability is shown in figure 5*c*. Here, we chose the minimum median development time of 70 h as this was approximately the lower end of the 95% confidence interval for the median maturation time at 10°C found in a large analysis of salmon farms in Norway [27], and thus is most likely the fastest that nauplii would develop into copepodites in the Broughton Archipelago. Again we can see that for certain median maturation times, the arrival probability is maximized at intermediate values of release farm position, *x*_0_, and therefore placing farms closer to each other may not always lead to higher transmission. However, for a given release farm position, the arrival probability is maximized at the lowest possible development time. Therefore, for two farms at fixed locations, warmer temperatures that cause faster louse development times will lead to higher spread between farms.

#### 3.4.4. How does cross-infection depend on the spacing between farms?

We can see that the degree of cross-infection between farms depends on a variety of factors, and there is no specific farm spacing that minimizes or maximizes cross-infection across all variables. If there is very little advection in the system then cross-infection will be highest when farms are closest together. In channels with an underlying advective current, cross-infection will be maximized at some intermediate spacing, though the spacing leading to maximum cross-infection depends on the current and development time.

## 4. Discussion

The degree of sea louse connectivity between salmon farms has been well studied in specific salmon farming regions, but there are few general models that can answer broad questions surrounding the effect of farm spacing and environmental variables on interfarm connectivity. In this paper, we used a simple mechanistic model to calculate the arrival time of sea lice dispersing between salmon farms and the level of cross-infection. We then calculated the same quantities directly from complex hydrodynamical and particle tracking simulations in the Broughton Archipelago and demonstrated that our simple model captures the necessary effects of environmental and physical variables on timing and cross-infection for sea lice in this region. Using our simple model calibrated to the Broughton Archipelago, we then investigated the effect of farm spacing, maturation time and ocean advection and diffusion on the degree of cross-infection between farms and found that there are several scenarios in which intermediate farm spacing leads to the highest levels of cross-infection.

In particular, if there is an underlying advective current in the channel, then the interfarm spacing that maximizes cross-infection will depend on the current speed. This is due in part to the maturation time required for sea lice to become infectious: if lice are swept by the farm before they have a chance to mature then cross-infection will be low, but if most mature before they arrive at the farm then cross-infection will be high. However, complicating this relationship is that for a given farm spacing, cross-infection may change throughout the year or among years as advection changes with river discharges and winds, as diffusion changes over spring-neap and longer tidal cycles, and as maturation time changes due to changing temperatures. Therefore, when considering farm placement in a salmon farming region, it is important to accurately measure the temperature of the ocean and average current speed between farms throughout the year to avoid placing farms at distances that will maximize cross-infection. Similarly when considering which farms to remove from a region, those at distances which maximize cross-infection to other farms should be strongly considered for removal.

It is the simple mechanistic nature of our model that allows us to explore the functional relationships between different parameters and understand how interfarm spacing affects cross-infection. By calibrating the model to hydrodynamic data from the Broughton Archipelago we ensure that our model accurately captures the essential components of ocean circulation and sea louse biology that govern lice dispersal between salmon farms. Studies that only use hydrodynamic models to investigate sea lice dispersal away from farms can accurately predict sea louse density in specific regions, but it is difficult for these studies to investigate the overall mechanistic relationships between the different parameters that govern sea louse spread between farms. By contrast statistical models can uncover the general relationships that govern spread between farms, but these relationships are often simple and linear and lack a mechanistic underpinning. While our simple mechanistic model has been calibrated to British Columbia data, if fitted to hydrodynamic data on sea louse dispersal between farms in a channel elsewhere we expect the parameter estimates to change, but we expect the functional relationship between the arrival time density and the various parameters in the model to remain the same. Therefore, the relationships uncovered here, for example how cross-infection between salmon farms in a channel can be maximized at an intermediate farm spacing, likely apply to other regions as well.

Previous studies from the Broughton Archipelago have also used hydrodynamic simulations as well as mechanistic models coupled with empirical data of sea lice counts on wild salmon to model the dispersal of sea lice away from salmon farms and these studies provide useful comparisons of parameter estimates in this region (table 1). All our parameter estimates are within the same range or of the same magnitude (when only one comparison is available) as those found previously, lending support to the accurate calibration of our simple model.

Another area in spatial ecology where intermediate, rather than extreme, habitat spacing can lead to increased transmission of larvae is in marine reserve design, but here the goal is to encourage dispersal between habitat patches, rather than prevent it. Ideally marine reserves, areas where there is typically no fishing or harvesting of a protected species, are large enough and sufficiently connected that they can sustain an entire connected metapopulation of one or many species, including the subpopulations not located in reserves. In coastal species with planktonic larval stages, the presence of an alongshore current can lead to most larvae settling tens of kilometres downshore from where they were released [44,45]. Therefore, when placing reserves in areas with an alongshore current, reserves should be spaced according to the mean advective distance of the current to maximize recruitment of larvae into the population [46]. When currents are strong, multiple evenly spaced reserves are more effective than a single large reserve and management based on marine reserves can outperform traditional effort-based management strategies [47]. Furthermore when realistic stochastic environmental variability is added to models with evenly spaced reserves with an alongshore current, a smaller total reserve area is required for the entire population to persist than would be predicted without environmental variability [48].

While an intermediate distance between habitat patches or salmon farms can maximize cross-infection in the presence of an advective current, when farms are not located in a channel but on nearby islands or along a common coastline the ocean dynamics will likely be diffusion dominated and thus cross-infection will likely decrease as farms become further apart. We found that our simple mechanistic model of arrival time also fits well to these scenarios (fits not shown), though in this case, the one-dimensional arrival rate (*α*) will likely be much lower. This is because the one-dimensional approximation of the channel or coastline is in the alongshore direction, and so it is assumed that any lice cross-shore of the farm can arrive at rate *α*. When both farms are in a channel dispersal is limited in the cross-shore direction by the edges of the channel, whereas on a coastline cross-shore dispersal is unbounded and so we expect a lower proportion of lice directly cross-shore of a farm to actually be capable of arriving on the farm. In cases where farms are along a common coastline or on nearby islands, we expect the probability of arrival to be well approximated by the seaway distance kernels used in other studies [25,27].

The simplicity of our mechanistic model coupled with the increases with respect to temperature also allows us to investigate how connectivity may change as ocean temperatures warm. We found that with the advective current in the Broughton Archipelago, shorter development times, which are caused by warmer temperatures, increased the probability of sea lice dispersing between salmon farms at fixed separation distances. This result lends support to previous work demonstrating that warmer temperatures increase the connectivity of farms in the Broughton Archipelago [41], and supports the theory that in 2015 anomalously warm temperatures contributed to a failure of salmon farms to control sea louse outbreaks, which led to high sea louse numbers on wild salmon [49]. Therefore, increased connectivity of salmon farms due to shorter development times of sea lice could lead to increased difficulty in controlling sea louse outbreaks on farms both in the Broughton Archipelago and in other salmon farming regions as ocean temperatures rise.

While our simple model can be used to understand how connectivity changes as temperatures warm, it may be beneficial to update connectivity estimates in future years. In this case, GPS drifters released from farms could be used to calculate the advection and diffusion of the ocean and determine spatial spread while average temperature and salinity data could be used to determine the appropriate maturation and survival times (equations (2.57)–(2.59)). GPS drifters have previously been used to determine ocean diffusivity [50,51], and could be used to update connectivity as re-running the hydrodynamic model is computationally expensive. In British Columbia, particle tracking models with sea louse releases from farms have only been run in the Broughton Archipelago and so drifters, combined with temperature and salinity data, could be used to estimate connectivity between salmon farms in other regions across British Columbia.

In this paper, we have estimated the timing and probability of arrival using advection and diffusion estimates from particle releases that have been averaged over 24 h to smooth the effect of the daily tidal cycle. However, there may be certain situations where the timing of arrival needs to be estimated at a specific point in the tidal cycle. It is also possible to account for the tidal cycle when calculating the arrival time density, though how exactly it is calculated depends on the assumptions that are made around sea lice movement during the tidal cycle. For completeness, we present both methods in the electronic supplementary material and compare the resulting arrival time densities to an arrival time density calculated directly using the hydrodynamical simulation with a single particle release. The first method allows the advection coefficient to oscillate in magnitude with the daily tidal cycle and in this case if the arrival time density is averaged over the tidal cycle it will be identical to the arrival time found in the main text. The second method builds on the first and assumes that sea lice can move between the main channel and small bays or connecting channels where they are free of the oscillating tidal flow. In this case the ability for lice to move between the main channel and small bays increases the effective diffusion that lice experience, and the arrival time found using this method no longer averages over the tidal cycle to the arrival time from the main text.

It should also be noted that in this paper we only use particle locations from a single particle release day in the hydrodynamic model to fit our arrival time density functions and find parameter estimates. The particular day, 2 May 2009, was the one chosen as being representative of the ‘average’ day by Cantrell *et al.* [10]. When the model is fitted to a higher connectivity day, shown in the electronic supplementary material, the estimates of advection and diffusion change, and if the model is fitted to data over many days it is likely that the advection coefficient will decrease in magnitude and the diffusion coefficient will increase [52]. In this paper, our goal of fitting the simple model to hydrodynamic data is to demonstrate that the simple mechanistic model can replicate realistic hydrodynamic scenarios. However, if the simple model is to be used to determine the ideal placement of salmon farms in a specific region, then the model should be fitted to hydrodynamic data throughout the year to accurately determine cross-infection between farms.

Lastly, this paper has been written in the context of the current agreement between the governments of British Columbia and the Kwikwasut’inuxw Haxwa’mis, ’Namgis and Mamalilikulla First Nations to remove salmon farms from their traditional territories [34]. Currently, nine farms are being removed before 2023 and after 2023 seven of the remaining 11 farms will require agreements with the Kwikwasut’inuxw Haxwa’mis, ’Namgis and Mamalilikulla First Nations and valid DFO licenses to continue to operate. Our work reinforces the notion that it is not always obvious how farm placement affects sea louse spread between farms, and we hope that our work can be used to better understand which farms may be the primary drivers of sea louse dispersal in the Broughton Archipelago and elsewhere.

## Data Availability

The data and code used to conduct the analyses reported in this paper are available through the Dryad Digital Repository: https://doi.org/10.5061/dryad.3j9kd51n0 [53]. Supplementary material is available online [54].
